# Mutations Driving Airborne Transmission of A/H5N1 Virus in Mammals Cause Substantial Attenuation in Chickens only when combined

**DOI:** 10.1038/s41598-017-07000-6

**Published:** 2017-08-03

**Authors:** Mathilde Richard, Sander Herfst, Judith M. A. van den Brand, Dennis de Meulder, Pascal Lexmond, Theo M. Bestebroer, Ron A. M. Fouchier

**Affiliations:** Department of Viroscience, Postgraduate School Molecular Medicine, Erasmus MC Rotterdam, The Netherlands

## Abstract

A/H5N1 influenza viruses pose a threat to human and animal health. A fully avian A/H5N1 influenza virus was previously shown to acquire airborne transmissibility between ferrets upon accumulation of five or six substitutions that affected three traits: polymerase activity, hemagglutinin stability and receptor binding. Here, the impact of these traits on A/H5N1 virus replication, tissue tropism, pathogenesis and transmission was investigated in chickens. The virus containing all substitutions associated with transmission in mammals was highly attenuated in chickens. However, single substitutions that affect polymerase activity, hemagglutinin stability and receptor binding generally had a small or negligible impact on virus replication, morbidity and mortality. A virus carrying two substitutions in the receptor-binding site was attenuated, although its tissue tropism in chickens was not affected. This data indicate that an A/H5N1 virus that is airborne-transmissible between mammals is unlikely to emerge in chickens, although individual mammalian adaptive substitutions have limited impact on viral fitness in chickens.

## Introduction

Influenza A viruses are enzootic in wild migratory aquatic birds around the world. Occasionally, these viruses spill over from this original reservoir - directly or via an intermediate host - into other animal hosts, including humans. Such zoonotic infections with avian influenza A virus occur relatively frequent^1^, but are generally restricted to sporadic individual cases. However, on rare occasions, zoonotic influenza A viruses can trigger a pandemic, i.e. a global outbreak caused by a new influenza A virus to which population immunity is low or absent. This happened four times in the last 100 years^2^. The key difference between zoonotic and pandemic influenza A viruses lies in their transmissibility. Whereas the former ones do not transmit sustainably among humans, the latter are transmitted via the airborne route, i.e via respiratory droplets and/or aerosols^2^.

Highly pathogenic avian influenza (HPAI) A/H5 viruses of the A/goose/Guangdong/1/96 (GsGd) hemagglutinin (HA) lineage have raised concerns about the possibility of a new pandemic^3^. These viruses are widespread in poultry in many countries across Eurasia and Africa and have devastated the poultry industry since the late 90 s. As a result of this continued circulation, they diversified in different genetic and antigenic clades, by accumulation of point mutations leading to distinct phenotypes^4^. Zoonotic transmission of HPAI GsGd A/H5 viruses from infected poultry can have fatal consequences. Since 2003, HPAI GsGd A/H5 viruses have caused 856 laboratory-confirmed human cases of infection, of which 452 persons died^5^. Whether HPAI GsGd A/H5 viruses could become transmissible among humans and mark the beginning of a new influenza pandemic has been one of the key questions of the last decades, notably because of the relatively high incidence and the severity of human infections with A/H5 viruses.

It is in this context that research on the requirements for HPAI A/H5 viruses of the GsGd lineage to become transmissible via the airborne route between mammals has been conducted, thereby identifying genetic and phenotypic traits associated with airborne transmission^6–10^. More specifically, Linster *et al*. identified a set of five substitutions, referred to as ‘airborne transmission substitutions’ throughout the rest of this manuscript, supporting the airborne transmissibility of a fully avian A/Indonesia/5/2005 (A/H5N1) virus (INDO)^10^: Q222L and/or G224S, H103Y and T156A in the HA gene (A/H5 numbering), E627K in the basic polymerase 2 (PB2) gene and H99Y in the basic polymerase 1 (PB1) gene. The HA-Q222L/G224S substitutions in the receptor binding site (RBS) of HA have previously been described to change the receptor binding preference of HAs of different subtypes from avian-type to human-type receptors, α2.3- and α2.6-linked sialic acids (SA) respectively^11–13^. The HA-T156A substitution, leading to the loss of a putative glycosylation site in the globular head of A/H5 HA, enhanced overall binding of HA to SA receptors^10^. The HA-H103Y substitution increased HA thermostability and pH stability and resulted in a reduced pH of HA-mediated membrane fusion^10, 14^. HA-mediated fusion of the viral and endosomal membranes occurs after receptor-mediated endocytosis of the virus, in order to release the viral genome in the cytoplasm. HA-mediated fusion takes place after HA has undergone an irreversible conformational change, triggered by acidification of the endosome. The PB2-E627K substitution in PB2 has been associated with increased replication of influenza viruses *in vitro* and *in vivo* at temperatures equivalent to those of the mammalian upper respiratory tract (URT)^15–17^ and with airborne transmission of 1918 A/H1N1 and A/H3N2 viruses^18, 19^. Finally, the PB1-H99Y substitution, in concert with PB2-E627K, tuned the balance between the expression levels of different RNA species (vRNA, mRNA, cRNA) transcribed by the viral polymerase complex, resulting in increased virus replication^10^.

The potential for airborne-transmissible avian-origin influenza viruses to evolve in a mammalian host has been described using mathematical modelling predicting that airborne substitutions could evolve within a single mammalian host, especially in an immunocompromised host^20, 21^. However, the likelihood of such viruses to emerge in their original hosts, i.e. poultry species, has yet to be determined. Exposure to poultry is the most likely route for humans to acquire an infection with avian influenza viruses and has been the source of many documented human cases of infection. Furthermore, although the genetic changes that have been described to promote airborne transmission of avian A/H5 viruses are the result of adaptation to mammalian hosts, some of these changes have also been detected in avian isolates. PB2-E627K has been found in 11% of the avian A/H5 viruses, as compared to 38% of the human A/H5 viruses^22^. Interestingly, HA-H103Y has been found in only 5 avian A/H5 strains but not in A/H5 human strains^22^. HA-T156A is present in 69% and 47% of the avian and human A/H5 strains respectively. Moreover, HA-T156A and HA-H103Y have been found in combination in 5 avian A/H5 viruses^22^. This suggests that the impact of -at least some of- the mammalian adaptive changes on viral fitness in avian hosts may be small, but our knowledge on this matter is virtually non-existent. Here, we studied the impact of (subsets of) HPAI A/H5N1 airborne transmission substitutions and related phenotypes on the replication, tissue tropism, pathogenesis and transmission of the INDO A/H5N1 virus in chickens.

## Results

### Airborne-transmissible A/H5N1 virus is attenuated in chickens

**Figure 1:**
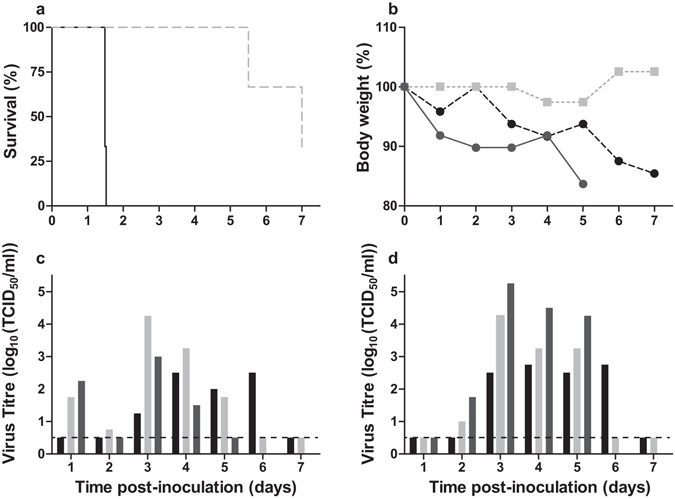
Survival, body weight and viral shedding of chickens upon inoculation with INDO_AT6_. (**a**) Survival (%) of chickens inoculated with INDO_WT_ (black line) or INDO_AT6_ (light grey dotted line). (**b**) Body weight (%) of chickens inoculated with INDO_AT6_. (**c**) Individual viral titres in oropharyngeal swabs collected from chickens inoculated with INDO_AT6_. (**c**) Individual viral titres in cloacal swabs collected from chickens inoculated with INDO_AT6_. In panels b, c and d, similar colour coding is used to identify individual animals. The lower limit of detection is indicated by the dashed lines.

To investigate the impact of a full set of six airborne transmission substitutions (HA-H103Y, HA-T156A, HA-Q222L, HA-G224S, PB1-H99Y and PB2-E627K) on viral replication, tissue tropism and pathogenesis in chickens, two groups of six 4 to 6 week-old chickens were inoculated with 10^5^ TCID_50_ of either the wild-type A/H5N1 virus (INDO_WT_) or the airborne-transmissible A/H5N1 virus (INDO_AT6_). Four hours post-inoculation (hpi), two chickens were added to each group of inoculated chickens to study transmissibility of the viruses (Figure S1). Chickens inoculated with INDO_WT_ succumbed to the infection at approximatively 36 hpi, which corresponded to 5 h to 10 h after the onset of symptoms (lethargy, ruffled feathers) (Fig. 1a and Table 1). In contrast, the mean time to death of the chickens inoculated with INDO_AT6_ was >6.25 days (Fig. 1a and Table 1). At 5 days post inoculation (dpi), one chicken presented neurological signs and was euthanized. At 7dpi, another chicken was found moribund. These chickens lost 16% and 15% of their starting bodyweight, respectively (Fig. 1b). The third chicken did not present any clinical signs until 7dpi. INDO_AT6_ was shed from both the oropharynx and the cloaca of all inoculated chickens (Fig. 1c,d and Table S1). In all three animals, the presence of all airborne substitutions in the last positive swabs with titres above 10^2^ TCID_50_/ml was confirmed (Table S2).

**Table 1:** Mean time to death of inoculated and contact chickens.

Viruses	Inoculated chickens	Contact chickens	
	Time to death (days)	Virus isolation/total	Time to death (days)
INDO_WT_	1.5/1.5/1.5	1/2	3.3/>7.0^a^
INDO_AT6_	5.5/7.0/>7.0	0/2	>7.0/>7.0
INDO_PB2-E627K_	1.4/1.5/1.9	0/2	>7.0/>7.0
INDO_PB1-H99Y_	1.0/1.4/1.5	2/2	2.8/3.8
INDO_HA-H103Y_	1.9/1.9/2.0	2/2	3.2/3.9
INDO_HA-T156A_	2.6/2.6/2.6	2/2	4.9/6.0
INDO_HA-Q222L/G224S_	2.4/2.4/2.5	0/2	>7.0/>7.0
INDO_HA-Q222L_	1.3/1.9/2.2	2/2	5.0/5.5
INDO_HA-G224S_	1.8/2.1/>7.0	0/2	>7.0/>7.0

^a^>7.0 means that animals did not get infected and were euthanized as the end of the experiment.

**Figure 2:**
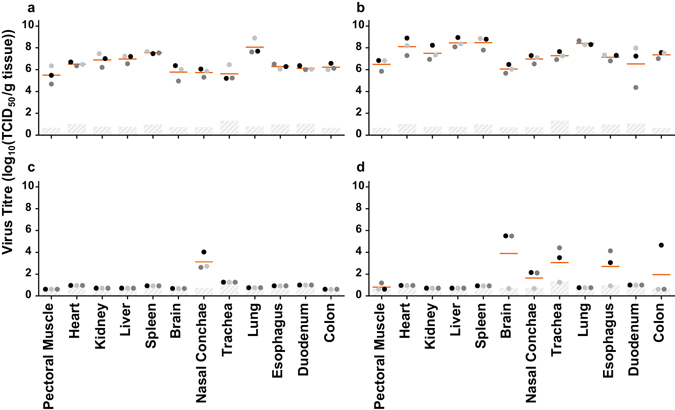
Viral titres in organs of chickens inoculated with INDO_WT_ or INDO_AT6_. (**a**) Individual viral titres in organs of chickens inoculated with INDO_WT_ at 24 hpi. (**b**) Individual viral titres in organs of chickens inoculated with INDO_WT_ at the time of death. (**c**) Individual viral titres in organs of chickens inoculated with INDO_AT6_ at 24 hpi. (**d**) Individual viral titres in organs of chickens inoculated with the INDO_AT6_ virus at the time of death. Lower limits of detection are indicated by the light grey bars. Means are indicated by the orange lines.

At 24 hpi, INDO_WT_ was detected at high titres in various organs (Fig. 2a). In all animals, the systemic spread of INDO_WT_ was associated with necrosis and inflammatory infiltrates in most organs that were examined, as well as with virus antigen expression as detected by immunohistochemistry (Table 2). However, no virus antigen was detected in the pancreas, pectoral muscle and brain. Virus antigen was primarily observed in endothelial cells and mononuclear cells in most positive tissues as well as in cardiomyocytes, hepatocytes and few epithelial cells of nose, trachea, lung, kidney, oesophagus, duodenum, colon and bursa. In contrast, INDO_AT6_ was only detected at lower titres in the nasal conchae, which was the site of inoculation (Fig. 2c). This correlated with the absence of lesions or virus antigen expression in all organs examined (Table 2).

**Table 2:** Virus antigen expression in tissues of chickens upon inoculation with INDO_WT_, INDO_AT6_, INDO_Q222L/G224S_, INDO_G224S_ and INDO_Q222L_.

Tissue	Virus antigen expression^a^ (no. virus antigen positive tissues/total no. of analysed tissues)					
	INDO_WT_	INDO_AT6_	INDO_AT6_	INDO_HA-Q222L/G224S_	INDO_HA-G224S_	INDO_HA-Q222L_
	24 hpi^b^	24 hpi	TOD^c^	24 hpi	24 hpi	24 hpi
Nasal Conchae	+(3/3)	—	—	+(2/3)	+(2/2)^d^	+(3/3)
Trachea	+(2/2)^d^	—	—	—	+(2/3)	+(2/3)
Lung	+(3/3)	—	—	+(1/3)	+(3/3)	+(3/3)
Heart	+(3/3)	—	—	—	+(1/3)	+(3/3)
Liver	+(3/3)	—	—	+(2/3)	+(3/3)	+(3/3)
Spleen	+(3/3)	—	—	+(2/3)	+(3/3)	+(3/3)
Kidney	+(3/3)	—	—	+(1/3)	+(2/3)	+(2/3)
Oesophagus	+(2/3)	—	—	—	+(3/3)	+(2/2)^d^
Proventriculus	+(2/3)	—	—	—	+(1/3)	+(1/2)^d^
Duodenum	+(2/3)	—	—	—	+(1/3)	—
Colon	+(3/3)	—	—	—	+(1/3)	+(2/2)^4^
Pancreas	—﻿	—	—	—	—	—
Mesenterium	+(1/3)	—	—	—	—	+(2/3)
Bursa	+(3/3)	—	—	—	+(2/3)	+(3/3)
Pectoral muscle	—	—	—	—	—	+(1/3)
Comb	+(2/3)	—	—	—	+(1/3)	+(3/3)
Brain	—	—	+(2/3)	—	—	+(2/3)

^a^Virus antigen expression was determined by immunohistochemistry targeting the influenza A nucleoprotein. +: virus antigen present, −: virus antigen not present.

^b^hpi: hours post-inoculation.

^c^TOD: Time of death.

^d^Assay performed on tissues from only two animals.

At the time of death, INDO_WT_ was detected in all collected organs, with titres that were on average one log higher than those at 24 hpi (Fig. 2b). In contrast, the INDO_AT6_ virus was detected in the respiratory tract (nasal conchae and trachea), intestinal tract (oesophagus and colon) and brain of the two chickens that became ill (Fig. 2d). Presence of replicating virus in the brain of these two chickens was confirmed by immunohistochemistry (Table 2), in which there was multifocal neuronal degeneration and mild necrosis with associated virus antigen in neurons, few glial cells and few ependymal cells but not in endothelial cells, as it was observed in the brains of chickens inoculated with INDO_WT_ (Figure S2). Interestingly, although the INDO_AT6_ bears a multi-basic cleavage site (MBCS) in HA, which allows the HA to be cleaved by ubiquitously expressed furin-like proteases^23^, the virus was not detected in extra respiratory tissues and intestinal organs other than the brain. No virus was detected, either by titration or by immunohistochemistry, in the tissues collected from the third animal at day 7, indicating that it had recovered from the infection. The presence of the INDO_AT6_ genotype was confirmed in all organs except for the nasal conchae tissues, for which amplification was not successful because of low titres, and for the trachea and oesophagus of one animal, for which low level of reversion to the wild-type genotype was detected at position 103 and 222 in HA (Table S2).

INDO_WT_ was transmitted to one of the contact chickens, which died 3 days after contact (Table 1). Virus titres in the organs were similar to those in the organs of chickens inoculated with the INDO_WT_ virus (Figure S3). In contrast, although INDO_AT6_ was shed up to 6 dpi, it was not transmitted to the contact chickens (Table 1 and Table S1). We conclude that INDO_AT6_ was severely attenuated in chickens compared to INDO_WT_, in terms of morbidity and mortality, virus replication, and tissue distribution.

### Combinatorial effect of the single airborne substitutions on the attenuated phenotype of the INDO _AT6_ virus

To investigate the contribution of each single airborne transmission substitution to viral phenotype in chickens, we performed similar experiments with viruses carrying single or double substitutions: INDO_HA-Q222L/G224S_, INDO_HA-Q222L_, INDO_HA-G224S_, INDO_HA-H103Y_, INDO_HA-T156A_, INDO_PB1-H99Y_ and INDO_PB2-E627K_. The mean time to death of chickens inoculated with INDO_PB2-E627K_ and INDO_PB1-H99Y_ were similar to that of chickens inoculated with INDO_WT_ (Table 1). Substitutions HA-H103Y, HA-T156A, HA-Q222L/G224S, HA-Q222L resulted in slightly delayed time to death (Table 1). One chicken inoculated with INDO_HA-G224S_ did not become infected and was euthanized at 7dpi (Table 1). INDO_PB1-H99Y_, INDO_HA-H103Y_, INDO_HA-T156A_ and INDO_HA-Q222L_ were transmitted to all contact chickens and transmission occurred within 2 to 6 days after exposure. In contrast, INDO_PB2-E627K_, INDO_HA-Q222L/G224S_ and INDO_HA-G224S_ were not transmitted to contact chickens (Table 1 and Figure S3).

**Figure 3:**
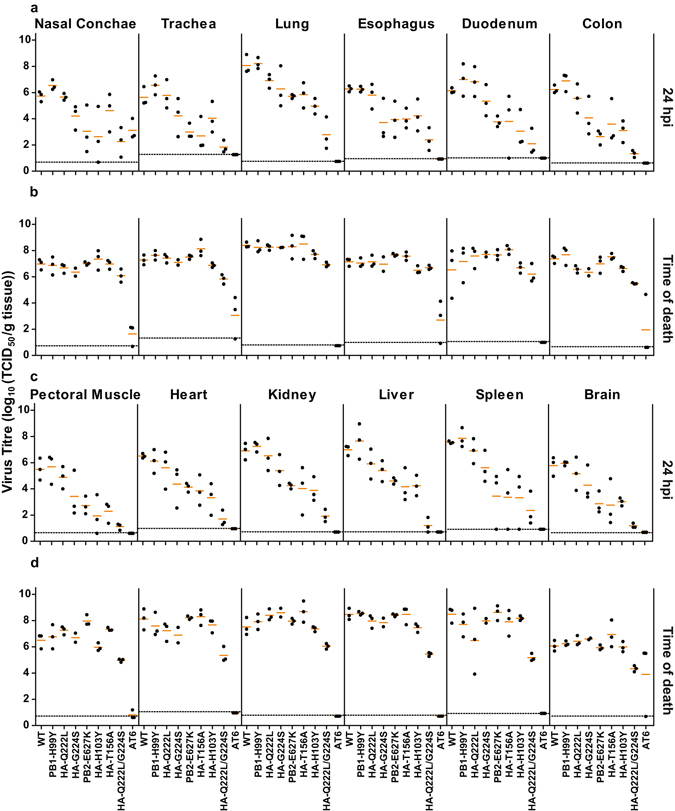
Viral titres in organs of chickens inoculated with mutant viruses. (**a** and **c**) Individual viral titres at 24 hpi in organs of chickens inoculated with mutant viruses carrying the indicated substitutions. (**b** and **d**) Individual viral titres at the time of death in organs of chickens inoculated with mutant viruses carrying the indicated substitutions. Lower limits of detection are indicated by the dotted lines. Mean virus titres are indicated by the orange lines.

At 24 hpi, all mutant viruses were detected in the vast majority of collected tissues, demonstrating that they were all able to spread systemically in chickens, even the viruses carrying the receptor binding substitutions HA-Q222L and/or HA-G224S. Interestingly, the level of attenuation caused by the different substitutions was consistent ﻿among organs, as demonstrated by similar titres for all three animals in all organs (Fig. 3). At 24 hpi, the titres in all organs of INDO_PB1-H99Y_ and INDO_HA-Q222L_-inoculated chickens were similar to those of INDO_WT_-inoculated chickens (Fig. 3a,c). The titres of INDO_HA-G224S_, INDO_HA-H103Y_, INDO_HA-T156A_, INDO_PB2-E627K_ and INDO_HA-Q222L/G224S_ were on average 1.7, 2.8, 2.9, 2.8 and 4.6 log lower as compared to those of INDO_WT_. At the time of death, only INDO_HA-Q222L/G224S_ was attenuated substantially, with titres on average 1.7 log lower than those of INDO_WT_ (Fig. 3b,d). Titres in the organs of contact chickens that became infected were similar to that of chickens inoculated with the respective viruses (Figure S3).

Viruses from the colon, lung and brain of each inoculated and virus-positive contact animal were sequenced to determine the presence or absence of the introduced substitutions (Table 3). No reversion to the WT genotype was detected in the investigated organs of inoculated and contact chickens of the INDO_PB2-E627K_, INDO_PB1-H99Y_, INDO_HA-T156A_ and INDO_HA-G224S_ groups. However, in all examined organs of one inoculated and one contact chicken of the INDO_HA-H103Y_ group, a mixed population of mutant and wild type amino acids was found at position 103 in HA. The highest level of reversion was observed with INDO_HA-Q222L_. In three out of six inoculated chickens, complete reversion to the WT genotype (Q) was detected in all investigated organs. In one other inoculated chicken, a mixture of wild type and mutant genotypes was detected. Moreover, virus isolated from one contact animal had a mixture of glutamine (Q) and arginine (R) at position 222 in all examined organs. When the HA-Q222L and the HA-G224S substitutions were combined, partial reversion to wild type genotype was only observed in one inoculated animal. Interestingly, for INDO_HA-Q222L/G224S_ at 24 hpi, the highest titres were found in the organs of the animal where reversion to wild-type genotype was detected.

**Table 3:** Presence of the introduced substitutions in tissues from chickens.

Virus	Tissue	Amino acid at the substituted position^a^							
		Inoc. 1				Inoc. 2		Contact	
		#1	#2	#3	#1	#2	#3	#1	#2
INDO_PB2-E627K_	Colon	K	K	K	K	K	K	n/a^b^	n/a
	Brain	K	K	K	K	K	K	n/a	n/a
	Lung	K	K	K	K	K	K	n/a	n/a
INDO_PB1-H99Y_	Colon	Y	Y	Y	Y	Y	Y	Y	Y
	Brain	Y	Y	Y	Y	Y	Y	Y	Y
	Lung	Y	Y	Y	Y	Y	Y	Y	Y
INDO_HA-H103Y_	Colon	Y	Y	Y	Y	Y	H/Y^c^	H/Y	Y
	Brain	Y	Y	Y	Y	Y	H/Y	H/Y	Y
	Lung	Y	Y	Y	Y	Y	H/Y	H/Y	Y
INDO_HA-T156A_	Colon	A	A	A	A	A	A	A	A
	Brain	A	A	A	A	A	A	A	A
	Lung	A	A	A	A	A	A	A	A
INDO_HA-Q222L/G224S_	Colon	L-S	L-S	L-S	L-S	L-S	Q/L-S	n/a	n/a
	Brain	L-S	L-S	L-S	L-S	L-S	Q/L -S	n/a	n/a
	Lung	L-S	L-S	L-S	L-S	L-S	Q/L -S	n/a	n/a
INDO_HA-Q222L_	Colon	Q	L	L	Q	Q/L	Q	L	Q/R
	Brain	Q	L	L	Q	Q/L	Q	L	Q/R
	Lung	Q	L	L	Q	Q/L	Q	L	Q/R
INDO_HA-G224S_	Colon	S	S	S	S	S	n/a	n/a	n/a
	Brain	S	S	S	S	S	n/a	n/a	n/a
	Lung	S	S	S	S	S	n/a	n/a	n/a

^a^The presence of the introduced substitution was investigated using Sanger sequencing.

^b^n/a: not applicable.

^c^Mixed population detected with Sanger sequencing.

Since the effect of substitutions in A/H5N1 HA that affect the receptor binding preference has not been studied in chickens extensively, we sought to study the pathogenesis and tropism of these viruses in detail. Histopathological examination and immunohistochemistry were performed on tissues from chickens inoculated with INDO_HA-Q222L/G224S_, INDO_HA-Q222L_ and INDO_HA-G224S_ and compared to tissues of chickens inoculated with INDO_WT_ at 24 hpi (Table 2). The chickens inoculated with INDO_HA-Q222L/G224S_ presented with only very limited lesions in nasal conchae, lung, liver and kidney except for multifocal necrosis and associated antigen presence in the spleen of one animal. Consistent with the data from the viral titrations, the lesions and associated antigen expression were more pronounced in the organs of the chickens inoculated with INDO_HA-G224S_ than in those inoculated with INDO_HA-Q222L/G224S_. The chickens inoculated with INDO_HA-Q222L_ had lesions and presence of virus antigen comparable to those in chickens inoculated with INDO_WT_, regardless of whether there was reversion to the WT genotype or not.

### The INDO_HA-Q222L/G224S_ virus has a similar attachment pattern to the chicken respiratory tract as the INDO_WT_ virus

**Figure 4:**
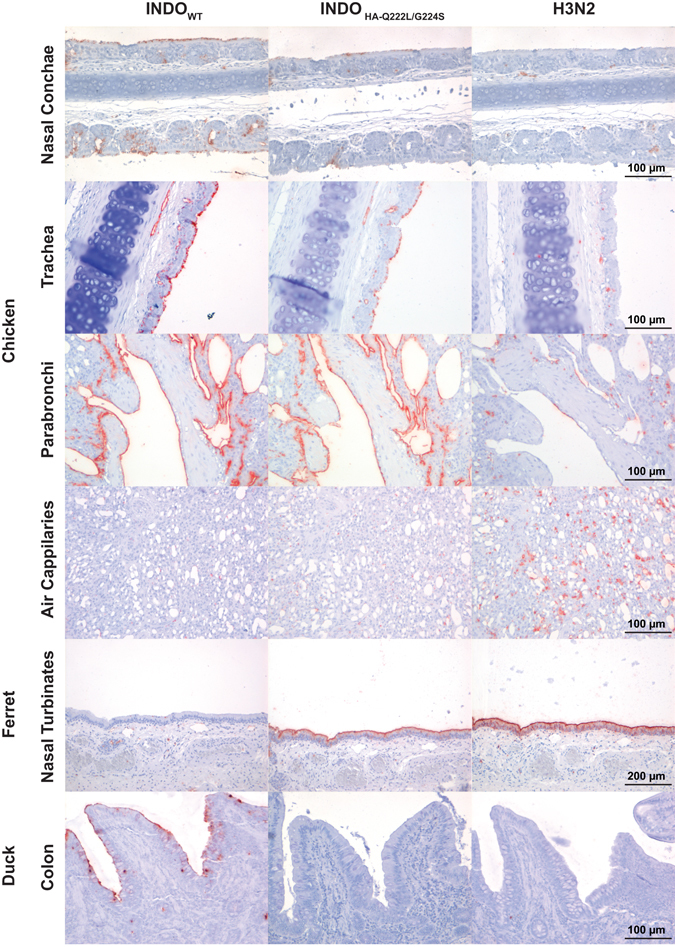
Attachment of INDO_HA-Q222L/G224S_ to different parts of the upper and lower chicken respiratory tract. The attachment of a human seasonal influenza virus A/H3N2 (A/Netherlands/213/2003) and of INDO_WT_ is shown for comparison. Every part of the chicken respiratory tract expresses both α.2,3-SA and α.2,6-SA. Attachment to ferret nasal turbinates and duck colon, which expresses α.2,6-SA and α.2,3-SA respectively, is shown as control for binding of human and avian influenza A viruses respectively.

The similarities in tissue tropism of INDO_WT_ and the viruses carrying the receptor binding substitutions were unexpected and prompted us to investigate the attachment pattern of these viruses to the chicken respiratory tract. We performed virus histochemistry on nasal conchae, trachea and lung tissues of two naïve animals (Fig. 4) and compared the attachment pattern of INDO_HA-Q222L/G224S_ to that of INDO_WT_ and a human A/H3N2 virus (A/Netherlands/213/2003). Unexpectedly, the attachment pattern of INDO_HA-Q222L/G224S_ was very similar to that of INDO_WT_ and different from that of the A/H3N2 virus. In the nasal conchae, INDO_WT_ attached focally to respiratory ciliated epithelial cells but more often than INDO_HA-Q222L/G224S_. Both INDO_HA-Q222L/G224S_ and INDO_WT_ attached to endothelial cells in large vessels and lumen of submucosal glands in the nasal conchae. In the trachea, both viruses attached abundantly and consistently to ciliated epithelial cells and to some endothelial cells of large vessels. In the lung, INDO_WT_ and INDO_HA-Q222L/G224S_ attached abundantly and consistently to the cuboidal epithelium of the parabronchi but not to the epithelium of the capillaries. In the lung, the only difference in attachment pattern between INDO_WT_ and INDO_HA-Q222L/G224S_ was that the mutant virus showed an intermediate level of attachment to cells of mononuclear appearance. On the other hand, the human A/H3N2 virus did not attach to the ciliated epithelium in the nose or in the trachea, attached occasionally and very weakly to the cuboidal epithelium of the parabronchi but abundantly to cells that morphologically resembled mononuclear cells in the nose, trachea and lung and occasionally to the endothelial cells in the capillaries.

## Discussion

Here we showed that an HPAI A/Indonesia/5/2005 (A/H5N1) virus carrying six substitutions that support airborne transmission between ferrets (INDO_AT6_) was highly attenuated in chickens. In contrast to INDO_WT_, which rapidly caused the death of all chickens in approximately 36 hpi, the INDO_AT6_-induced disease progressed slowly and eventually two out of three animals succumbed to the infection at 5 and 7 dpi respectively. The third chicken shed virus up to 6 dpi and had recovered by 7 dpi from the infection. Furthermore, whereas INDO_WT_ spread systematically and was isolated from all sampled organs, which is the hallmark of HPAI infection in poultry species^24, 25^, INDO_AT6_ was only detected in the respiratory and intestinal tract and in the brain of two animals at the time of death. Given the fact that the only extra-respiratory and intestinal organ in which INDO_AT6_ was detected is the brain, where only neurons, glial cells and ependymal cells were virus antigen positive, INDO_AT6_ most probably spread to the brain via the olfactory route, rather than the vascular route. Moreover, contrary to INDO_WT_, which was detected in endothelial cells throughout the body, including in the brain at the time of death, INDO_AT6_ was not detected in any endothelial cells in the brain. Therefore, although INDO_AT6_ carries a MBCS, it did not spread systemically, probably because of the attenuation or a change in endothelium tropism caused by the airborne transmission substitutions. This confirms that a MBCS in HA is not necessarily sufficient for systemic spread of HPAI viruses^26^.

The attenuated phenotype of INDO_AT6_ is likely due to a combinatorial effect of multiple airborne substitutions. Individual substitutions caused different levels of attenuation - ranging from almost no attenuation to a high level attenuation -, but with none of the single substitutions resulting in attenuation similar to that conferred by INDO_AT6_. Overall, the time to death was slightly delayed and viral titres at 24 hpi were lower compared to INDO_WT_, but the virus tropism of all mutant viruses was similar to that of INDO_WT_. Moreover, whereas the INDO_AT6_ virus was not transmitted to co-housed chickens, transmission was observed for INDO_WT_ and some of the mutant viruses: INDO_PB1-H99Y_, INDO_HA-H103Y_, INDO_HA-T156A_ and INDO_HA-Q222L_.

PB2-E627K has been identified as a major determinant of host adaptation of pandemic influenza viruses. In our experiments, substitution PB2-E627K resulted in moderate attenuation of the INDO_WT_ virus in chickens and no reversion to the WT genotype was detected in the organs that were analysed. It was previously shown that viruses carrying a lysine at position 627 are not attenuated as compared to wild-type viruses in avian cells lines^27–29^. In chickens, it was shown that the effect of the PB2-E627K substitution on A/H5N1 virus fitness was dependent on the virus lineage^29^. Clade 2.2 A/H5N1 viruses, which were first detected in the outbreak of Qinghai Lake in China^30^ and subsequently spread to Europe, Southern Asia and Africa, already possess a lysine at position 627 in PB2 and this genotype has been maintained in viruses of this lineage until present^31^. In this virus lineage, no differences in fitness in *in vitro* and *in vivo* studies in avian hosts have been observed between viruses bearing an E or a K at position 627 in PB2^29, 32^. In contrast, when the PB2-E627K substitution was introduced in a 1995 HPAI A/H5N1 virus, reversion to 627E was observed in experimentally infected chickens^29^. The reason for these apparent virus lineage-dependent effects of PB2-E627K on the virus in chickens is unknown, but constraints at the RNA level could be implicated^29^. A recent study shed light on the mechanism of host range restriction by identifying a host factor, ANP32, which interacts with influenza polymerase^33^. E627K enables avian polymerase activity to be supported by short versions of ANP32, typical of mammalian hosts. However, chickens possess both long (ANP32A) and short version (ANP32B), enabling both human and avian polymerase activities to be supported, suggesting a minimal impact of humanized polymerase in avian hosts.

Substitution PB1-H99Y did not attenuate the INDO virus in chickens. This substitution was found to compensate the effect of PB2-E627K on the balance between vRNA, cRNA and mRNA produced during infection of mammalian cells^10^. This substitution was neutral in chicken, probably because of the little impact of E627K on polymerase function in chickens.

INDO_HA-H103Y_ virus was also slightly attenuated in chickens and partial reversions to the WT genotype were detected in one inoculated and one contact chicken, suggesting this substitution is not well tolerated in chickens. The HA-H103Y substitution has been described to increase HA acid stability by lowering the pH at which the HA-mediated fusion is triggered, and to increase HA thermostability^10^. HA stability has been described to be an important host-range factor and to play a role in inter- and intraspecies adaptation and transmission^34^. An analysis of HAs from 16 different subtypes showed that the pH of HA activation of almost all HAs of humans isolates was lower (i.e. a more stable HA) than that of avian isolates from the same subtype^35^. Furthermore, low pH of HA activation has been associated with airborne transmissibility of avian viruses in the ferret model^9, 10^, increased replication in the upper respiratory tract of mammals^36^ and also with pandemic potential of pandemic A/H1N1 viruses^37^.

There is a trade-off between the intracellular and extracellular stability of HA. A virus with an unstable HA, which will undergo the conformational change required for membrane fusion at a relatively high pH, will release its genome in the cytoplasm from early endosomes to escape lysosomal degradation. However, this virus will also be prone to inactivation in acidic or mildly acidic extracellular space (in the host or in the environment). On the other hand, a virus with an HA that is highly stable will be stable in (acidic) environments^38^ but will be prone to lysosomal degradation. The attenuation in chickens of the INDO virus by HA-H103Y is consistent with a study describing the correlation between increased pathogenicity of an A/H5N1 virus in chickens and increased pH of HA activation^39^. Interestingly, although the HA-H103Y substitution was found to be detrimental in our experiment, which correlates with published literature, the HA-H103Y substitution has been identified in five A/H5 avian isolates^22^. One of these viruses was an HPAI A/H5N1 strain from the GsGd lineage, A/Duck/Zhejiang/BJ/2002, in which the HA-T156A was also detected. Moreover, the HA-H103Y substitution was detected during passaging of a wild duck A/H5N1 LPAI virus in chicken embryos^40^.

HA stability and pH of HA activation do not only have a role in replication in different hosts/tissues but also play a role in transmission. In wild bird species, viruses are transmitted via the faecal-oral route and shared water. In these species, one could hypothesize that environmental survival is an important factor for propagation of the virus^41^. On the other hand, in poultry species that are housed in dry and denser households, environmental stability might not be as critical. There is some evidence that poultry adapted viruses HPAI viruses are less stable than LPAI viruses^42, 43^. However, the comparison of related A/H7N3 duck and turkey influenza virus isolates revealed two amino acids differences in the HA responsible for lowering the pH of activation in the turkey viruses^44^. In ducks, it has been shown that transmissibility was increased by substitutions that slightly reduced the pH of activation leading to higher shedding and greater environmental persistence^38^. Decreased pH of fusion was also found critical for airborne transmissibility of A/H9N2 between chickens^45^, but it still remains unclear what are the main routes of transmission of LPAI and HPAI viruses in poultry flocks, contact, dust-borne or airborne. Knowledge on the endosomal pH and extracellular pH of different tissues in various hosts and environments should help to understand the species and virus differences in terms of pH of activation.

INDO_HA-T156A_ was also attenuated, but no reversion to the wild type genotype was detected. Amino acid 156 in HA is located in the proximity of the RBS, at the tip of the globular head. Attenuation of INDO_HA-T156A_ correlates with studies showing that acquisition of additional glycosylation in the globular head of HA is a common evolutionary change as the result of adaptation to poultry species^46, 47^. However, the impact of the HA-T156A substitution might be context- or species dependent. Many avian HPAI GsGd A/H5 strains of clade 2.2 and 2.3, including the latest clade 2.3.4.4^48^ indeed possess this substitution. A phylogenetic analysis showed that clade 2.2 viruses in Egypt carrying the HA-T156A substitution first emerged in birds and were subsequently transmitted to humans^31^, suggesting that in these lineages, this substitution is well tolerated.

The largest attenuation effect was found in mutant viruses harbouring the receptor binding site mutations that change the receptor binding preference towards human-type receptors. INDO_HA-Q222L/G224S_ was the most attenuated mutant virus, although the mean time to death was only delayed by one day and the tropism of the virus was not affected. For the single mutant viruses, the viral titres in organs were lower for INDO_HA-G224S_ than for INDO_HA-Q222L_. However, the titres of the INDO_HA-Q222L_ might not completely reflect the phenotype of the HA-Q222L substitution as reversions were detected in both inoculated and contact chickens. In the samples that we analysed, full reversion to WT genotype was detected in three inoculated chickens and partial reversion in one inoculated chicken, suggesting that the HA-Q222L substitution was less stable than HA-G224S in chickens. Interestingly, in one contact chicken, an arginine was detected at position 222. The receptor binding properties conferred by this substitution in the A/H5 HA in unknown, but it has been demonstrated that this substitution abrogates α2.6-linked SA binding of A/pH1N1 HA completely^49^. Notably, this substitution has been detected in a human isolate from Cambodia as a mixed population with Q222^50^.

The HA-Q222L/G224S substitutions have been associated with the establishment of the A/H2N2 and A/H3N2 pandemic viruses in humans and with a switch from α2.3- to α2.6-linked SA receptors preference in these and several other HA subtypes^11, 13^. In A/H5N1, the Q222L/G224S double substitution was responsible for a decrease in α2.3-linked SA receptor binding and an increase in binding to α2.6-linked SA receptors^12, 51^. Moreover, the binding pattern of an A/H5N1 virus carrying the HA-Q222L/G224S to human and ferret respiratory tract tissues was similar to that of a human A/H3N2 virus^12^. Here we show that the attachment pattern of the INDO_HA-Q222L/G224S_ to the respiratory tract of chicken was similar to that of the INDO_WT_ and different from that of a human A/H3N2 virus. Studies using lectin staining or virus histochemistry have shown that, contrary to waterfowl species in which α2.3-linked SA are predominantly found, epithelial cells in the respiratory tract and intestinal tract of chicken harbour both α2.3- and α2.6-linked SA receptors^52–56^. Moreover, influenza viruses from terrestrial poultry differ from waterfowl influenza viruses by their ability to bind α2.6-linked SA^57^, suggesting that land-based poultry could act as possible intermediates for the generation of viruses with dual receptor binding preference and pandemic potential. The presence of both α2.3- and α2.6-linked SA in chickens might explain why both the INDO_WT_ and INDO_HA-Q222L/G224S_ viruses were able to attach to epithelial cells throughout the respiratory tract. This correlates with the observation that INDO_WT_ attached abundantly to the duck colon epithelium, where α2.3-linked SA receptors are predominantly present, whereas INDO_HA-Q222L/G224S_ did not. However, although both INDO_HA-Q222L/G224S_ and the human A/H3N2 virus attached to α2.6-linked SA^12^ in *in vitro* assays, they did not attach to similar sialylated structures on chicken respiratory tissues. In other assays where more complex sialylated structures are also present, such as glycan arrays, the attachment pattern of INDO_HA-Q222L/G224S_ was also not similar to that of human A/H1N1 viruses^14^. Despite similar binding patterns to the chicken respiratory tract, INDO_HA-Q222L/G224S_ was still attenuated compared to INDO_WT_, possibly because of binding to different glycans and/or with different avidity, which cannot be assessed by using virus histochemistry. No avian A/H5N1 viruses with both the HA-Q222L and the HA-G224S substitutions have been detected in nature. However, the HA-Q222L was found as a mixed population with Q222 in one human A/H5N1 strain from Cambodia, and also in combination with an HA-N220K substitution^50^. This HA-Q222L/N220K double substitution has been associated with airborne transmission of a virus carrying an avian A/H5^9^. Moreover, the HA-Q222L substitution has been detected in other avian strains of the A/H7 and A/H9 subtypes^2, 58^.

It has been shown that several other substitutions in HA, identified in virus isolates or in laboratory experiments, have an equivalent receptor binding phenotype as the HA-Q222L and HA-G224S substitutions^59^. Several other amino acids substitutions that increase binding to α2.6-linked SA, without necessarily completely abrogating binding to α2.3-linked SA receptors, have been identified in HPAI GsGd A/H5 human isolates of different clades, maybe as the result of adaptation to humans^60–64^. However, a phylogenetic study showed that substitutions that enabled α2.6-linked SA binding of clade 2.2 viruses from 2006 to 2009 in Egypt were actually acquired during their circulation in birds^64^. These viruses were also still able to bind α2.3-linked SA receptors and to replicate in avian cells, which is probably necessary for efficient transmission among birds.

Based on the level of attenuation of INDO_AT6_, we conclude that the likelihood of such a combination of substitutions to emerge in chickens in the specific viral backbone that we studied here is very low. In the unlikely event that such combination of substitutions would be selected in chickens, the virus would still have to be efficiently transmitted among chickens to pose a potential threat. Although the prolonged survival and shedding of chickens inoculated with INDO_AT6_ might favor transmission to contact chickens or mammals, INDO_AT6_ virus was not transmitted to direct contact chickens. Other parameters than the duration or level of shedding influencing transmission efficacy include stability in the environment (in feces, water, dust or air) as well as the infectious dose necessary to infect a naïve animal. It would also be interesting to assess whether INDO_AT6_ could be better transmitted to mammals than INDO_WT_ by performing chicken to ferret airborne transmission^65, 66^.

Although a full set of airborne-transmissible substitutions is unlikely to emerge in chickens, individual phenotypes supported by these substitutions had a limited impact on the virus in chickens. Humanized polymerase and increased HA stability could be supported without major fitness loss in avian hosts. Receptor binding change did not change the tissue tropism of the virus in chickens. Moreover, functionally equivalent substitutions might have a less attenuated phenotype in avian species. Therefore, a continuous monitoring for the early detection of human adaptation substitution in avian influenza viruses, as part of pandemic preparedness programs, is warranted.

## Methods

### Cells

Madin-Darby canine Kidney (MDCK) cells (ATCC) were cultured in Eagle’s minimal essential medium (EMEM, Lonza Benelux BV, Breda, the Netherlands) supplemented with 10% fetal bovine serum (FBS) (Greiner), 100 U/ml penicillin (P, Lonza), 100 U/ml streptomycin (S, Lonza), 2 mM L-glutamine (L-glu, Lonza), 1.5 mg/ml sodium bicarbonate (NaHCO_3_, Lonza), 10mM Hepes (Lonza) and 1X non-essential amino acids (NEAA, Lonza). 293T cells were cultured in Dulbecco modified Eagle’s medium (DMEM, Lonza) supplemented with 10% FBS, 100 U/ml P, 100 U/ml S, 2 mM L-glu, 1mM sodium pyruvate (Gibco) and 1X NEAA.

### Viruses

The gene segments of A/Indonesia/5/2005 (A/H5N1) virus (clade 2.1) containing the substitutions HA-H103Y, HA-T156A, HA-Q222L, HA-G224S, HA-Q222L/G224S, PB1-H99Y and PB2-E627K, were produced as previously described^10^. Recombinant viruses (INDO_WT_, INDO_PB2-E627K_, INDO_PB1-H99Y_, INDO_HA-H103Y_, INDO_HA-T156A_, INDO_HA-Q222L_, INDO_HA-G224S_, INDO_HA-Q222L/G224S_ and INDO_AT6_) were produced upon transfection of 293T cells as described previously^67^. Viruses were propagated one time in MDCK cells to generate a stock and titrated in MDCK cells as described below. The presence of the desired substitution(s) and the absence of any other substitutions examined in this study were verified using Sanger sequencing in each virus stock.

### Chicken experiment

Animals were housed and experiments were conducted in strict compliance with European guidelines (EU directive on animal testing 86/609/EEC) and Dutch legislation (Experiments on Animals Act, 1997). All animal experiments were approved by the independent animal experimentation ethical review committee ‘stichting DEC consult’ (Erasmus MC permit number EUR3385). Research projects involving laboratory animals can only be executed if they are approved by the DEC. The DEC considers the application and pays careful attention to the effects of the intervention on the animal, its discomfort and weighs this against the social and scientific benefit to humans or animals. The researcher is required to keep the effects of the intervention to a minimum, based on the three Rs (Refinement, Replacement, Reduction).

One-day-old specific-pathogen-free (SPF) female White Leghorn chickens were received from Gezondheidsdienst voor Dieren B.V. (Deventer, Netherlands) and housed at the Erasmus Dierexperimenteel Centrum until the age of 4 to 6 weeks. Nine groups of six chickens were inoculated intranasally with 10^5^ TCID_50_ of virus (INDO_WT_, INDO_PB2-E627K_, INDO_PB1-H99Y_, INDO_HA-H103Y_, INDO_HA-T156A_, INDO_HA-Q222L_, INDO_HA-G224S_, INDO_HA-Q222L/G224S_ or INDO_AT6_). Four hpi, two naive contact chickens per group were placed in the same isolator as the inoculated chickens. Daily, the bodyweight of inoculated and contact chickens was monitored and oropharyngeal and cloacal swabs were collected and stored at −80 °C in transport medium (Hank’s balanced salt solution containing 10% of glycerol, 200 U/ml P, 200 mg/ml S, 100 U/ml polymixin B sulphate (Sigma) and 250 mg/ml gentamicin (Gibco)) until end-point titration in MDCK cells. Twenty four hpi, 3 inoculated chickens per group (Inoc. 1) were euthanized by exsanguination under anaesthesia (ketamine/medetomidine). Pectoral muscle, heart, liver, spleen, pancreas, kidney, brain, nasal conchae, trachea, lung, oesophagus, duodenum and colon were harvested, homogenized in transport medium using the FastPrep system (MP Biomedicals) with 2 one-quarter-inch ceramic sphere balls, centrifuged 1500 × g for 10 min, aliquoted and stored at −80 °C for endpoint titration in MDCK cells. Pieces of these organs, along with the comb, proventriculus, gizzard and bursa of Fabricius were fixed in formalin for immunohistochemistry and pathology (see below). The course of the infection was followed until 7 dpi/contact for the three other inoculated chickens (Inoc. 2) and the two contact chickens of each group. Euthanasia was performed if the animals lost more than 15% of their bodyweight in two days or 20% in the whole experiment and according to the severity of the disease. The experimental design is summarized in Figure S1.

### Virus titration in MDCK cells

MDCK cells were inoculated with tenfold serial dilutions of virus stocks, oropharyngeal swabs, cloacal swabs and homogenized tissue samples as previously described^48^.

### Sanger Sequencing

Viral RNA was extracted from virus stocks, swabs and organs using the High Pure RNA Isolation Kit (Roche). Gene segments of influenza virus were amplified by RT-PCR^68^ and sequenced using a BigDye Terminator v3.1 Cycle sequencing kit (Applied Biosystems, Nieuwekerk a/d IJssel, the Netherlands) and a 3130XL genetic analyser (Applied Biosystems), according to the instructions of the manufacturer.

### Pathology and Immunohistochemistry

After fixation in 10% neutral-buffered formalin, tissues were embedded in paraffin, sectioned at 3 μm and stained with hematoxylin and eosin (HE) for the detection of histological lesions by light microscopy. For the detection of virus antigen by immunohistochemistry, tissues were stained with a monoclonal antibody against influenza A virus nucleoprotein as the primary antibody as described previously^69^. Tissues from a non-inoculated chicken were included in the study as a negative control. Immunohistochemical and pathological analysis were performed blindly by a veterinary pathologist. One tissue section was analyzed per tissue. All the fields available on each tissue section were analyzed. Pictures were taken using an Olympus BX51 microscope and acquisition Olympus Cell^A^ software.

### Virus Histochemistry

The pattern of attachment of the viruses to chicken respiratory tract tissues was determined by virus histochemistry as described previously^70^. Briefly, formalin-fixed, paraffin-embedded tissue sections from two naive animals were deparaffinised in xylene and hydrated using graded alcohols. Fluorescin isothiocyanate (FITC)-labelled influenza viruses (INDO_WT_, INDO_HA-Q222L/G224S_ and A/Netherlands/213/2003 (A/H3N2, human isolate propagated in MDCK) were incubated overnight at a concentration of 50 hemagglutination units/50 μL. For visualization by light microscopy, FITC was detected with a peroxidase-labelled rabbit–anti-FITC (DAKO, Glostrup, Denmark). The signal was amplified using a tyramide amplification system (Perkin-Elmer, Boston, MA). Peroxidase was revealed with 3-amino-ethyl-carbozyle (Sigma-Aldrich) resulting in a bright red precipitate. Control tissues, ferret nasal turbinates and duck colon, were included as controls for binding of human and avian viruses respectively. Pictures were taken using an Olympus BX51 microscope and acquisition Olympus Cell^A^ software.

### Biosafety

All experiments were conducted within the enhanced animal biosafety level 3 (ABSL3+) facility of Erasmus MC. The ABSL3+ facility consists of a negative pressurized (−30Pa) laboratory in which all *in vivo* and *in vitro* experimental work is carried out in class 3 isolators or class 3 biosafety cabinets, which are also negative pressurized (<−200Pa). Although all experiments are conducted in closed class 3 cabinets and isolators, special personal protective equipment, including laboratory suits, gloves and FFP3 facemasks is used. Air released from the class 3 units is filtered by High Efficiency Particulate Air (HEPA) filters and then leaves via the facility ventilation system, again via HEPA filters. Only authorized personnel that have received the appropriate training can access the ABSL3+ facility. For animal handling in the facilities, personnel always work in pairs. The facility is secured by procedures recognized as appropriate by the institutional biosafety officers and facility management at ErasmusMC and Dutch and United States government inspectors.

### Data availability

All data generated during and/or analysed during the current study are available from the corresponding author on reasonable request.

## Electronic supplementary material

Supplementary Information
