# Supplementary material for: Mutations Driving Airborne Transmission of A/H5N1 Virus in Mammals Cause Substantial Attenuation in Chickens only when combined
Source: Sci Rep. 2017 Aug 3;7:7187. doi: 10.1038/s41598-017-07000-6 (PMC5543172; doi:10.1038/s41598-017-07000-6)
Supplement: Supplementary file 1 — Supplementary Information [file 41598_2017_7000_MOESM1_ESM.pdf]

Mutations Driving Airborne Transmission of A/H5N1 Virus in Mammals Cause Substantial Attenuation in Chickens only when combined

Mathilde Richard*, Sander Herfst, Judith M.A. van den Brand, Dennis de Meulder, Pascal Lexmond, Theo M. Bestebroer and Ron A.M. Fouchier.

Table S1. Viral titres in swabs of inoculated and contact chickens. Related to Figure 1 and Figure 3.

Virus	Group	Virus titre ($log_{10}(TCID_{50}/ml)$)													
		Day 1		Day 2		Day 3		Day 4		Day 5		Day 6		Day 7	
		OS[a]	CS[b]	OS	CS	OS	CS	OS	CS	OS	CS	OS	CS	OS	CS
INDO WT	Inoc. 1	3	2,5	-	-	-	-	-	-	-	-	-	-	-	-
		4,25	1,5	-	-	-	-	-	-	-	-	-	-	-	-
		3	1,25	-	-	-	-	-	-	-	-	-	-	-	-
	Inoc. 2	2,75	2,5	-	-	-	-	-	-	-	-	-	-	-	-
		3,25	0,5[c]	-	-	-	-	-	-	-	-	-	-	-	-
		2,5	0,5	-	-	-	-	-	-	-	-	-	-	-	-
	Contact	0,5	0,5	0,5	0,5	0,5	0,5	0,5	0,5	0,5	0,5	0,5	0,5	0,5	0,5
		0,5	0,5	0,5	0,5	2,25	3,25	-	-	-	-	-	-	-	-
INDO AT6	Inoc. 1	2,25	0,5	-	-	-	-	-	-	-	-	-	-	-	-
		3,25	0,5	-	-	-	-	-	-	-	-	-	-	-	-
		1,75	0,5	-	-	-	-	-	-	-	-	-	-	-	-
	Inoc. 2	0,5	0,5	0,5	0,5	1,25	2,5	2,5	2,75	2	2,5	2,5	2,75	0,5	0,5
		1,75	0,5	0,75	1	4,25	4,5	3,25	3,25	1,75	3,25	0,5	0,5	0,5	0,5
		2,25	0,5	0,5	1,75	3	5,25	1,5	4,5	0,5	4,25	-	-	-	-
	Contact	0,5	0,5	0,5	0,5	0,5	0,5	0,5	0,5	0,5	0,5	0,5	0,5	0,5	0,5
		0,5	0,5	0,5	0,5	0,5	0,5	0,5	0,5	0,5	0,5	0,5	0,5	0,5	0.5
INDO PB2-E627K	Inoc. 1	4	1,5	-	-	-	-	-	-	-	-	-	-	-	-
		2	0,5	-	-	-	-	-	-	-	-	-	-	-	-
		3,5	1	-	-	-	-	-	-	-	-	-	-	-	-
	Inoc. 2	3	0,5	-	-	-	-	-	-	-	-	-	-	-	-
		3,25	0,5	-	-	-	-	-	-	-	-	-	-	-	-
		4,5	1,5	-	-	-	-	-	-	-	-	-	-	-	-
	Contact	0,5	0,5	0,5	0,5	0,5	0,5	0,5	0,5	0,5	0,5	0,5	0,5	0,5	0,5
		0,5	0,5	0,5	0,5	0,5	0,5	0,5	0,5	0,5	0,5	0,5	0,5	0,5	0,5
INDO PB1-H99Y	Inoc. 1	4	3,5	-	-	-	-	-	-	-	-	-	-	-	-
		3,5	1,25	-	-	-	-	-	-	-	-	-	-	-	-
		4,25	2,25	-	-	-	-	-	-	-	-	-	-	-	-
	Inoc. 2	4,75	0,5	-	-	-	-	-	-	-	-	-	-	-	-
		5,5	5,25	-	-	-	-	-	-	-	-	-	-	-	-
		4	2,5	-	-	-	-	-	-	-	-	-	-	-	-
	Contact	0,5	0,5	0,5	0,5	-	-	-	-	-	-	-	-	-	-
		0,5	0,5	0,5	0,5	3	2,75	-	-	-	-	-	-	-	-
INDO HA-H103Y	Inoc. 1	0,5	0,5	-	-	-	-	-	-	-	-	-	-	-	-
		4,5	1,5	-	-	-	-	-	-	-	-	-	-	-	-
		5,25	1,25	-	-	-	-	-	-	-	-	-	-	-	-
	Inoc. 2	4,25	1,5	-	-	-	-	-	-	-	-	-	-	-	-
		4,25	1	-	-	-	-	-	-	-	-	-	-	-	-
		5,25	0,5	5	4,75	-	-	-	-	-	-	-	-	-	-
	Contact	0,5	0,5	1	0,5	0,5	0,5	3,5	2	-	-	-	-	-	-
		0,5	0,5	1	0,5	3,25	3,5	-	-	-	-	-	-	-	-
INDO HA-T156A	Inoc. 1	4,75	1,25	-	-	-	-	-	-	-	-	-	-	-	-
		2,25	0,75	-	-	-	-	-	-	-	-	-	-	-	-
		2	0,5	-	-	-	-	-	-	-	-	-	-	-	-
	Inoc. 2	3,75	1	ND[d]	ND	-	-	-	-	-	-	-	-	-	-
		3	0,5	ND	ND	-	-	-	-	-	-	-	-	-	-

		2	1	ND	ND	-	-	-	-	-	-	-	-	-	-
	Contact	0,5	0,5	0,5	0,5	0,5	0,5	0,5	0,5	0,5	0,5	5	6	-	-
		0,5	0,5	0,5	0,5	0,75	0,5	2	3,5	-	-	-	-	-	-
INDO HA-Q222L/ G224S	Inoc. 1	1	1	-	-	-	-	-	-	-	-	-	-	-	-
		2,25	1	-	-	-	-	-	-	-	-	-	-	-	-
		1,25	0,5	-	-	-	-	-	-	-	-	-	-	-	-
	Inoc. 2	2,25	1	3,25	3,25	-	-	-	-	-	-	-	-	-	-
		1,75	2,5	3	1,5	-	-	-	-	-	-	-	-	-	-
		2,75	2,25	3,25	1,5	-	-	-	-	-	-	-	-	-	-
	Contact	0,5	0,5	0,5	0,5	0,5	0,5	0,5	0,5	0,5	0,5	0,5	0,5	0,5	0,5
		0,5	0,5	0,5	0,5	0,5	0,5	0,5	0,5	0,5	0,5	0,5	0,5	0,5	0,5
INDO HA- Q222L	Inoc. 1	3,75	4,25	-	-	-	-	-	-	-	-	-	-	-	-
		1,75	2,25	-	-	-	-	-	-	-	-	-	-	-	-
		3,5	1	-	-	-	-	-	-	-	-	-	-	-	-
	Inoc. 2	3,25	3,5	-	-	-	-	-	-	-	-	-	-	-	-
		2	0,75	-	-	-	-	-	-	-	-	-	-	-	-
		3,75	0,5	-	-	-	-	-	-	-	-	-	-	-	-
	Contact	0,5	0,5	3,5	0,5	0,5	0,5	0,75	1,5	-	-	-	-	-	-
		0,5	0,5	1,75	0,5	0,5	0,5	0,75	0,75	1,5	0,5	-	-	-	-
INDO HA-G224S	Inoc. 1	3,5	3,75	-	-	-	-	-	-	-	-	-	-	-	-
		4	2	-	-	-	-	-	-	-	-	-	-	-	-
		3	2,25	-	-	-	-	-	-	-	-	-	-	-	-
	Inoc. 2	5,25	0,5	4,25	4,75	-	-	-	-	-	-	-	-	-	-
		0,5	0,5	0,5	0,5	0,5	0,5	0,5	0,5	0,5	0,5	0,5	0,5	0,5	0,5
		2,5	3,5	ND	ND	-	-	-	-	-	-	-	-	-	-
	Contact	0,5	0,5	0,5	0,5	0,5	0,5	0,5	0,5	0,5	0,5	0,5	0,5	0,5	0,5
		0,5	0,5	0,5	0,5	0,5	0,5	0,5	0,5	0,5	0,5	0,5	0,5	0,5	0,5

[a] OS: Oropharyngeal Swab

[b] CS: Cloacal Swab

[c] 0.5 represent the limit of detection of the assay.

[d] ND: Not Determined

Table S2. Sanger sequencing of swabs and organs of chickens inoculated with the $INDO_{AT6}$ virus. Related to Figure 1 and 2.

Chicken	Sample	Amino acid at the indicated position					
		PB2	PB1	HA	HA	HA	HA
		627	99	103	156	222	224
	Inoculum	K	Y	Y	A	L	S
Chicken 1	Oropharyngeal Swab Day 6	K	Y	Y	A	L	S
	Cloacal Swab Day 6	K	Y	ND[a]	A	L	S
Chicken 2	Oropharyngeal Swab Day 5	K	Y	Y	A	L	S
	Cloacal Swab Day 5	K	Y	Y	A	L	S
Chicken 3	Oropharyngeal Swab Day 3	K	Y	Y	A	L	S
	Cloacal Swab Day 5	K	Y	Y	A	L	S
Chicken 1	Brain	K	Y	Y	A	L	S
	Trachea	K	Y	Y/H	A	L	S
	Esophagus	K	Y	Y/H	A	L/Q	S
	Colon	K	Y	Y	A	L	S
Chicken 3	Brain	K	Y	Y	A	L	S
	Trachea	K	Y	Y	A	L	S
	Esophagus	K	Y	Y	A	L	S

[a] ND : Not Determined

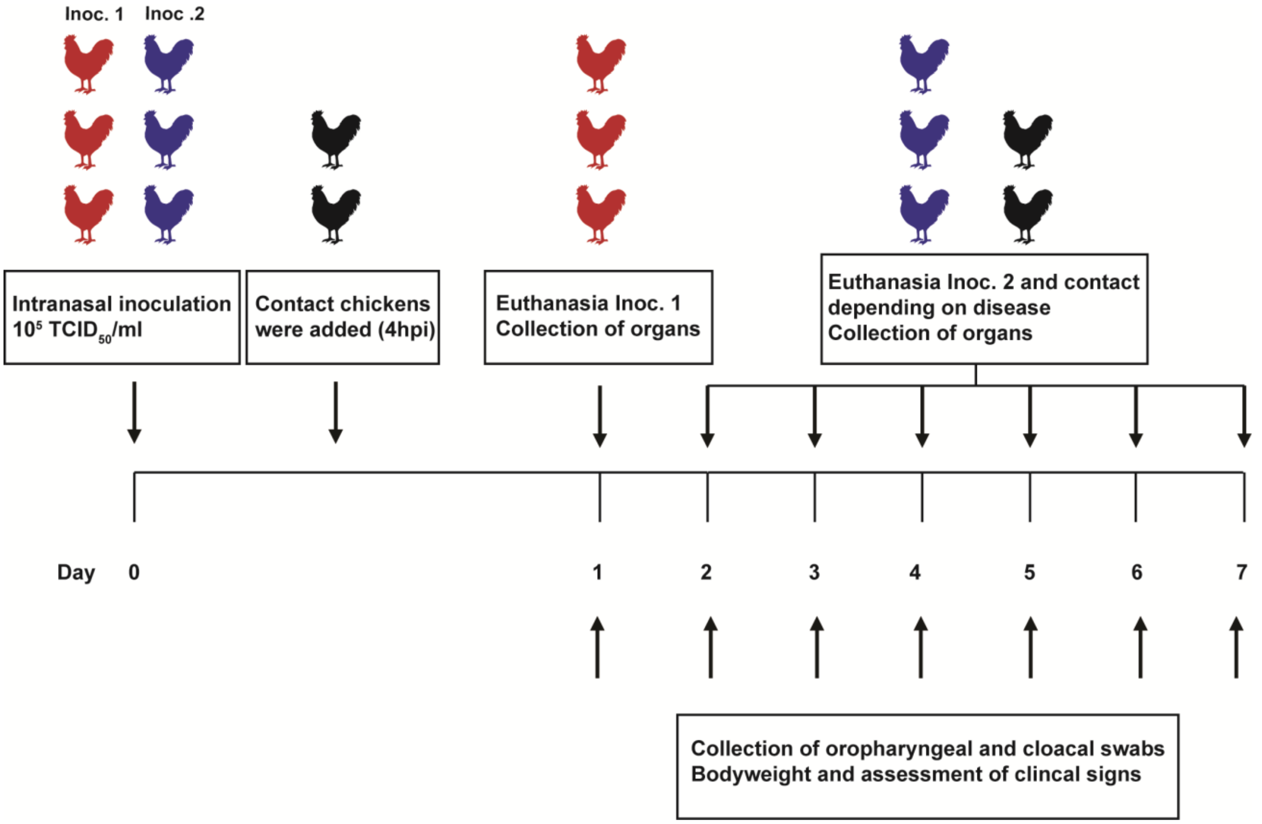

Supplementary Figure 1. Experimental design of the chicken experiments.

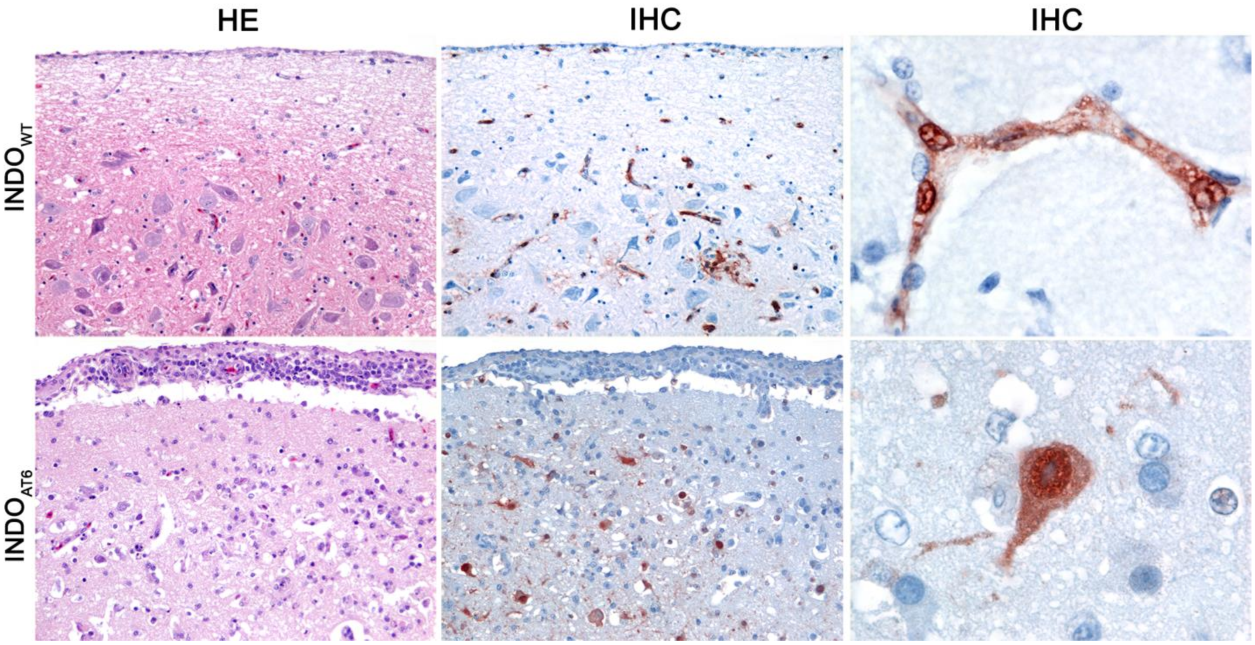

Supplementary Figure 2. Histopathology and immunohistochemistry of the brain of chickens inoculated with INDO$_{WT}$ or INDO$_{AT6}$ virus at time of death. Representative images of hematoxylin-eosin (HE) staining for histopathology and immunohistochemistry (IHC) for detection of influenza A virus nucleoprotein antigen are depicted. In the brain of chickens inoculated with INDO$_{WT}$, there was multifocal mild gliosis and moderate necrosis associated with virus antigen in glial cells and also antigen present in many endothelial cells. In the brain of chickens inoculated with INDO$_{AT6}$, there was severe lymphocytic infiltration in the meninges and perivascular space (cuffing), multifocal neuronal necrosis, edema and gliosis with associated virus antigen expression in neurons and few glial cells.

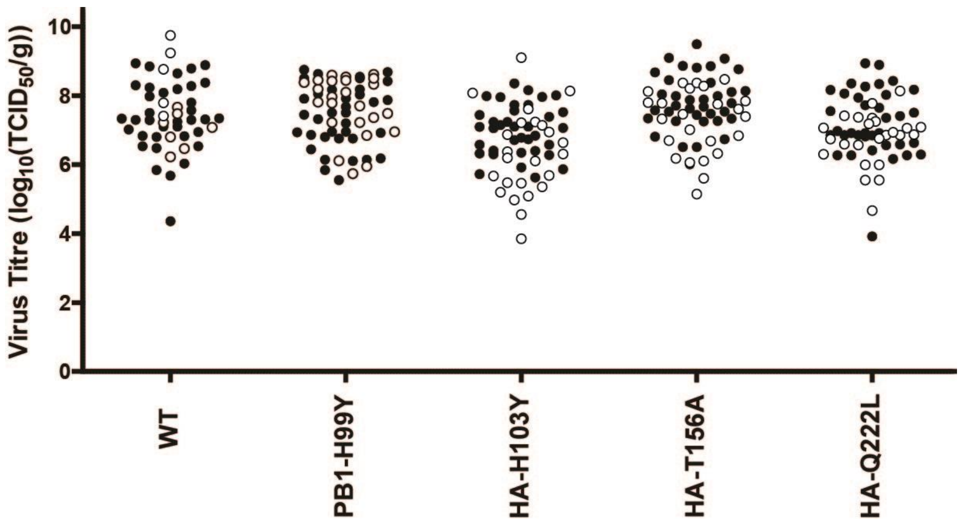

Supplementary Figure 3. Comparison between the viral titres retrieved from organs of inoculated and contact chickens. Black dots represent titres from individual organs of chickens inoculated with viruses carrying the indicated substitution at the time of death. Open dots represent viral titres from individual organs of contact chickens at the time of death. Titres from organs of inoculated and contact chickens are within the same range. Related to Table 1.
